# Effect of Local and General Anesthetics on Interfacial Water

**DOI:** 10.1371/journal.pone.0152127

**Published:** 2016-04-07

**Authors:** Nenad Kundacina, Minghui Shi, Gerald H. Pollack

**Affiliations:** Department of Bioengineering, University of Washington, Seattle, Washington, United States of America; University of California, Merced, UNITED STATES

## Abstract

**Background:**

Water undergoes structural change as it interfaces with hydrophilic surfaces, including the many hydrophilic surfaces within the cell. This interfacial water has become known as “Exclusion Zone (EZ) water” or “fourth-phase water” [1].

**Methods:**

We tested the hypothesis that anesthetics diminish the amount of EZ water, and that this change may correlate with functional changes in anesthesia. By using the local anesthetics Lidocaine and Bupivacaine as well as a general inhalational anesthetic, Isoflurane, we tracked the EZ size as these anesthetics were introduced.

**Results:**

All three anesthetics diminished EZ size in a concentration-dependent manner at concentrations of 0.18 mM and greater for Bupivacaine, 0.85 mM and greater for Lidocaine, and 0.2% for Isoflurane. At extremely low (micromolar) concentrations, however, all three anesthetics increased EZ size.

**Conclusions:**

The sharp increase of EZ size associated with micromolar anesthetic concentrations follows a similar pattern to induction of general anesthesia, from the excitation stage (Stage II) to the depression and overdose stages of surgical anesthesia (Stages III and IV). The results are consistent with the hypothesis that anesthetics may act on water, a fundamental organizational component common to all cells.

## Introduction

The mechanism by which anesthetics block function remains incompletely understood. The first modern theory was proposed by Hans Meyer and again by Charles Overton [2, 3]. According to that theory, anesthetics diffuse into the lipid bilayer of cell membranes. This changes the cell’s chemistry by affecting the solubility of ions and other molecules in fatty substances. Meyer tested the solubility of various molecules and ions in olive oil, which was meant to represent fatty molecules in brain cells. He compared olive oil’s solubility with and without anesthetics added. He then tested the anesthetics on tadpoles to observe any behavioral effects. While his tadpole experiments were imprecise and subjective, Meyer was able to correlate lipid solubility with anesthetic concentration [2]. Overton conducted experiments similar to Meyer’s and was also able to correlate lipid solubility with the concentration of anesthetic [3].

Similar ideas followed those original proposals. For example, Miller and colleagues proposed that anesthetic accumulation in the membrane bilayer reduced the effectiveness of channel proteins which in turn prevented proper ion flow [4]. However, other investigators raised objections: Drugs that are soluble in lipids would be expected to have a similar effect, but do not. Hydrophobic molecules such as halogenated alkanes fail to induce narcosis [5]. Temperature increases would also be expected to increase membrane fluidity and thus increase the anesthetic effect, but this effect is not seen [6]. Nevertheless, most investigators today consider that anesthetics act on membrane proteins, channels, or receptors, with current theories emphasizing membrane lateral pressures, and the models of protein binding originally introduced by Franks and Lieb [6].

Linus Pauling proposed an entirely different idea much earlier: that anesthetics stabilized hydrate structures, preventing normal electrical signals and ion flow and thereby inducing narcosis [7]. Pauling’s idea lacked direct evidence, and was apparently not followed up.

Recently, it has become clear that cells contain considerable amounts of interfacial water [8]. Most water molecules lie within one nanometer of some hydrophilic surface; therefore, cell water is mainly interfacial water. Studies of interfacial water—now termed “exclusion zone” (EZ) water because of its profound solute-exclusionary nature—have shown that EZ water builds extensively next to hydrophilic surfaces, including gels and biological surfaces [8]. Given the abundance of EZ water in the cell, the question arose whether EZ water might be a possible target of anesthetic action.

Recent evidence has given force to this hypothesis. A threshold amount of water needs to be present near hydrophilic protein sites in order for anesthetics to work effectively [9]. It is reasonable then to think that anesthetics might interact in some way with water to produce narcosis.

One particular hydrophilic material that creates a prominent exclusion zone is the polymer Nafion. Nafion has a Teflon backbone with many anionic sulfonate groups attached, so it resembles a cellular membrane with a negative charge. It can thus bond to water. Nafion has been used in many studies of EZ properties, and was used as a model for the current experiments.

The aim of this work was to determine if there is a relationship between water’s EZ and anesthesia’s mechanism of producing narcosis. The results confirmed this expectation.

## Experimental Procedures

### Chamber

**Fig 1:**
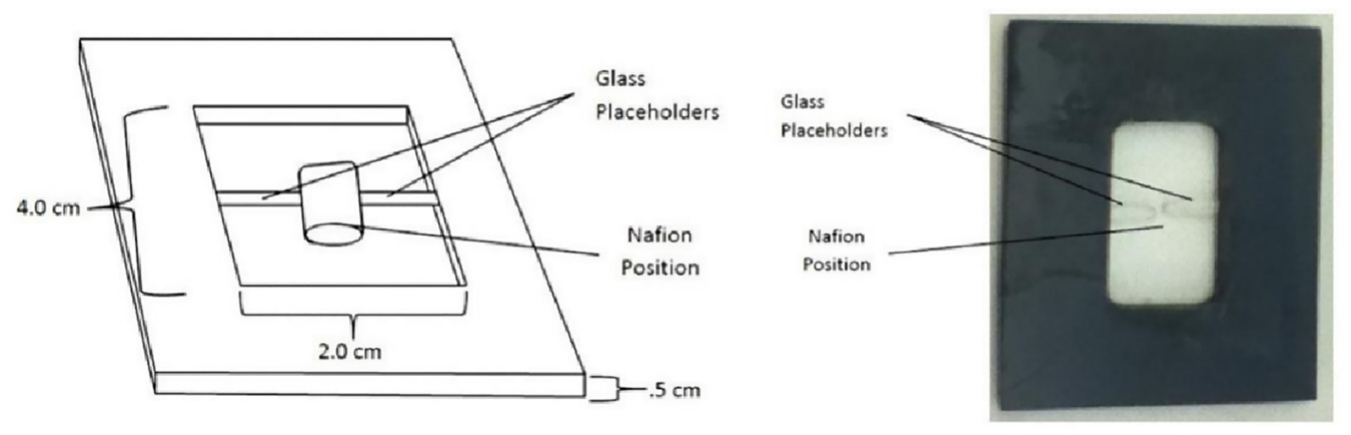
Chamber schematic (left) and top view photo (right).

The test chamber used for experimentation was a 4 x 2 x 0.5 cm custom-made unit composed entirely of glass (Fig 1). The bottom was sealed with transparent glass while the top was open to the air. Two glass placeholders positioned on the bottom of the chamber were used for holding the Nafion tube firmly in place. The Nafion tube, usually about 1 cm long and 0.25 cm in diameter, was positioned in between the two placeholders.

### Water

Deionized water was obtained from a NANOpure Diamond ultrapure water system. The purity of water from this system was certified by a resistivity value up to 18.2 MΩ · cm, which exceeds ASTM, CAP, and NCCLS type-I water requirements. In order to define the far edge of the exclusion zone, microspheres were added to the water to create a suspension. The suspension was prepared by filling a 20 mL tube with deionized water and adding two drops of PolyScience Inc. Polybead 1-micron carboxylate microspheres.

For each trial, the chamber was filled with 2.5 mL of the microsphere suspension. A 1-cm long Nafion tube was then placed as shown in Fig 1. In order to prevent the tube from floating, the tube was first filled with the microsphere suspension, using a pipette. This prevented air bubbles from being trapped within the tube and thereby causing the tube to float.

### Bupivacaine and Lidocaine

**Fig 2:**
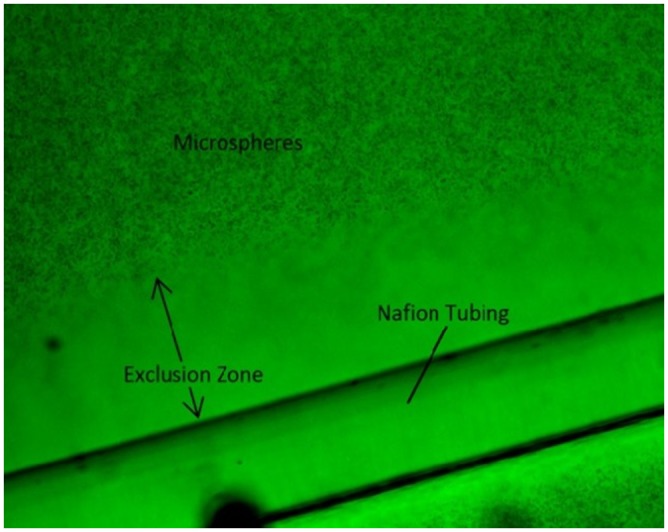
Exclusion Zone image, obtained with ImageJ. Green filter was used to minimize microscope illumination.

After the Nafion tube had been secured, the chamber was placed on the stage of a Zeiss Axiovert 35 inverted fluorescence microscope and observed with a 5x objective lens. Observations were made at room temperature (21–23°C), and microscopic illumination was kept constant in order to avert any light-induced changes. The exclusion zone was seen forming along the edge of the Nafion, as shown in Fig 2. Images were recorded using ImageJ software. The exclusion zone took about 5 minutes to fully form. Once it had reached a stable size, the anesthetic was added to the chamber using a pipette. Two local anesthetics used at varying concentrations, 0.5% Bupivacaine (APP Pharmaceuticals) and 2% Lidocaine (APP Pharmaceuticals), were each added in 10 μL, 25 μL, 50 μL, and 100 μL doses to the chamber. This results in concentrations of .02 μg/mL, .05 μg/mL, 0.1 μg/mL and 0.2 μg/mL for Bupivacaine and .08 μg/mL, 0.2 μg/mL, 0.4 μg/mL and 0.8 μg/mL for Lidocaine. These ranges of concentrations include the clinically relevant concentrations of 0.1 μg/mL for Bupivacaine and 0.2 μg/mL for Lidocaine that are used for epidural injections [10, 11]. Using ImageJ, an image was captured every thirty seconds. Once the exclusion zone had stabilized at its new value, its size could be measured and recorded over multiple frames.

### Isoflurane

Similar procedures were followed for Isoflurane (Sigma-Aldrich). After the Nafion tube had settled at the bottom of the chamber, 2 mL of microsphere suspension was added. The chamber was then covered with a clean glass slide and placed on the microscope stage. EZ formation along the outer edge of the Nafion tube was observed with a 5x objective lens.

Ten minutes were allowed for the EZ to stabilize at room temperature (21–23°C). The width of EZ was then measured with the software ImageJ and recorded. The chamber was then emptied except for the Nafion tube, rinsed with deionized water, and lightly dried with a paper towel. The anesthetic was then applied in varying doses.

To apply the anesthetic, the following procedures were used. 2 mL of microsphere suspension was poured into a clean plastic test tube. After adding 2 μL of liquid Isoflurane with a pipette, the tube was capped and swirled gently by hand so that the anesthetic was well mixed with the microsphere suspension. The mixed suspension was added to the chamber, which was then covered with a glass slide to prevent evaporation. The chamber was then returned to the microscope stage for visualization. Measurement procedures were the same as for the other anesthetics.

Those steps were repeated with Isoflurane concentrations ranging from 0.1% to 0.6% (v/v). These Isoflurane concentrations correspond to alveolar percent concentrations ranging from .4% to 2.4%. The clinically relevant Minimum Alveolar Concentration (MAC), which corresponds to 50% response to stimuli, is around 1.15% for Isoflurane, depending on the individual [12]. For each new measurement, the Nafion tube used for the previous run was discarded and a new one used.

### Reversibility

In order to test for reversibility, Bupivacaine was added to Nafion in solution in the same manner as before. Once the exclusion zone was diminished and measured, the solution was gradually replaced with fresh solution. This was done by using a pipette to gradually remove 50 μL of anesthetic microsphere suspension from the test chamber and replace it with the same amount of suspension that had no anesthetics added. After 0.5 mL of solution was replaced, the exclusion zone was measured once more.

## Results

**Fig 3:**
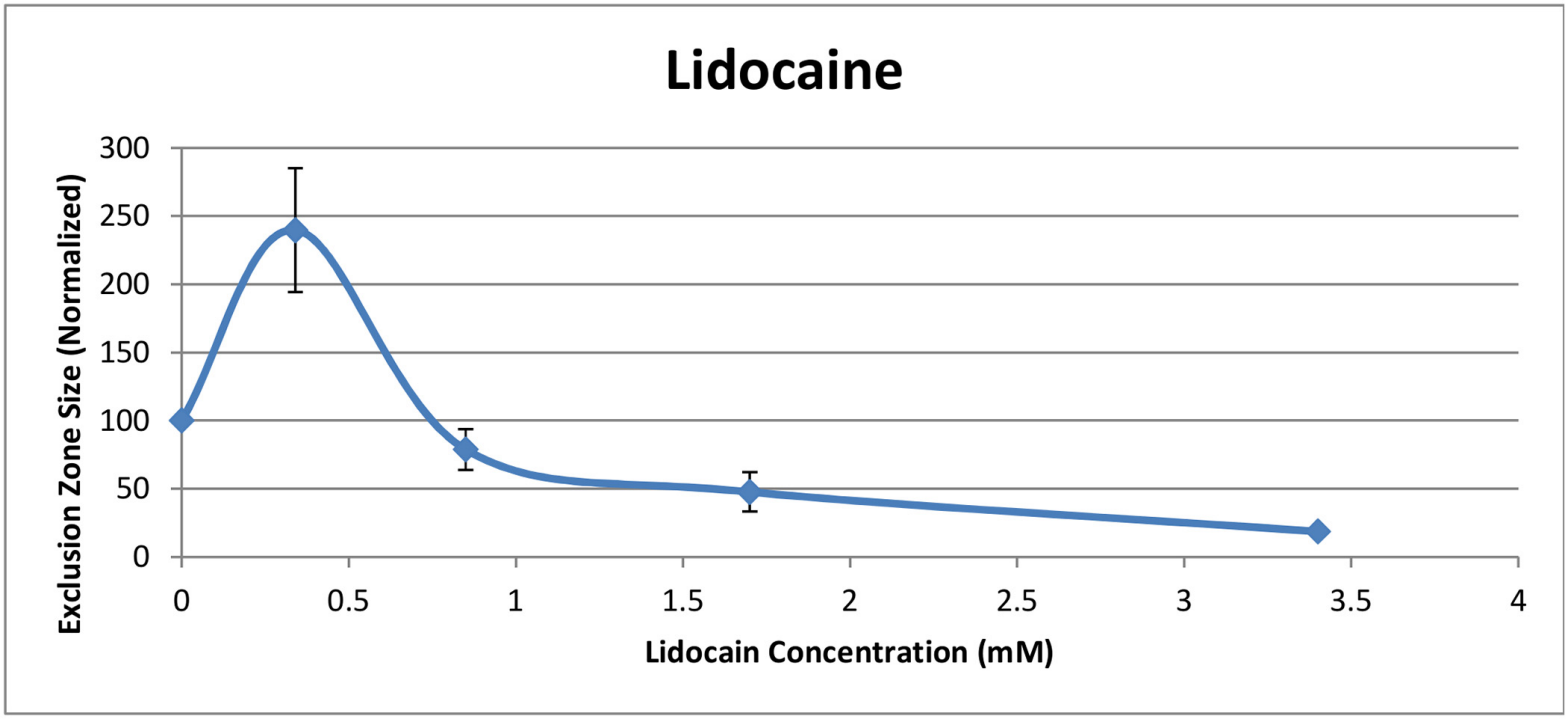
Exclusion zone size (normalized) after addition of Lidocaine.

**Fig 4:**
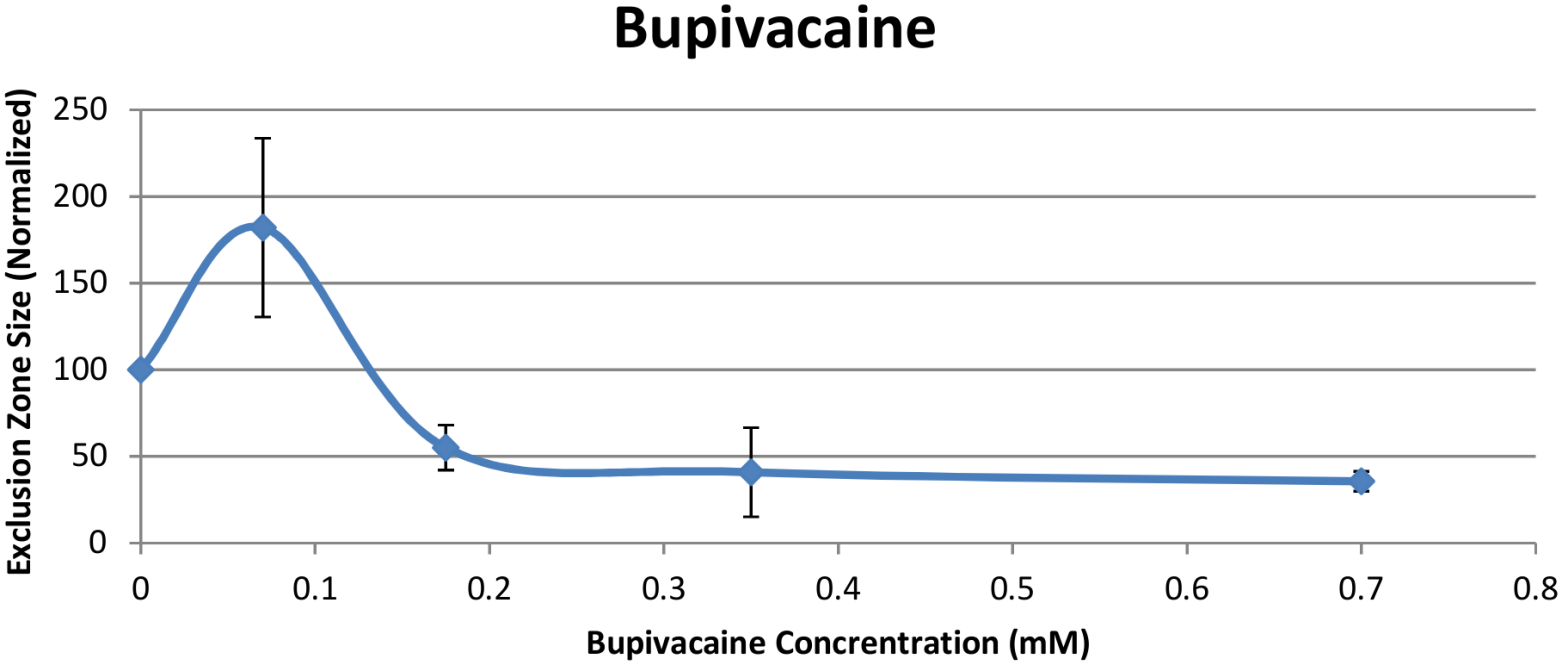
Exclusion zone size (normalized) after addition of Bupivacaine.

All concentrations of local anesthetics affected the exclusion zone significantly. This was true of both Lidocaine (Fig 3) and Bupivacaine (Fig 4). In general, EZ size diminished with higher anesthetic concentrations.

A surprising result occurred at the lower concentrations of both Lidocaine and Bupivacaine. At 0.34 mM Lidocaine and 0.07 mM Bupivacaine, the exclusion zone expanded substantially. While the standard deviation of this value was relatively high, the standard deviation divided by the mean value was similar to other values (Tables 1 and 2). Hence, the trials were fairly consistent over the full range of concentrations.

**Table 1:** Summary of Bupivacaine Results.

Volume Bupivacaine (μL)	Concentration (mM)	Initial EZ (Normalized)	Final EZ (Normalized)	ST.DEV	Number of Trials	Final / St. Dev.
0	0	100	100	0	5	N/A
10	0.07	100	182	51.5	5	3.5
25	0.18	100	55.1	13.0	5	4.2
50	0.35	100	40.9	25.7	5	1.6
100	0.70	100	35.7	5.7	5	6.2

“Normalized” values refer to actual values in micrometers divided by experimental values, also in micrometers.

**Table 2:** Summary of Lidocaine Results.

Volume Lidocaine (μL)	Concentration (mM)	Initial EZ (Normalized)	Final EZ (Normalized)	ST.DEV	Number of Trials	Final / St. Dev.
0	0	100	100	0	5	N/A
10	0.34	100	239	45.5	5	5.3
25	0.85	100	78.8	15.0	5	5.3
50	1.7	100	47.7	14.4	5	3.3
100	3.4	100	18.6	2.2	5	8.5

**Fig 5:**
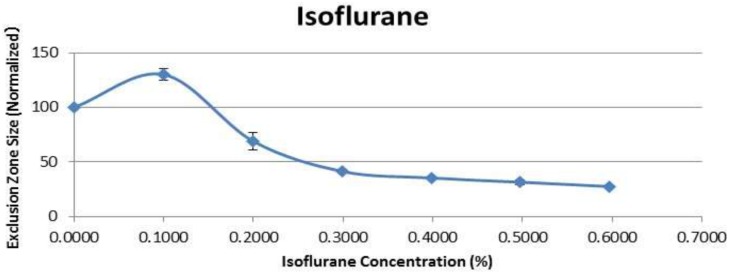
Exclusion zone size (normalized) after addition of Isoflurane.

Much like the local anesthetics, the general anesthetic Isoflurane increased EZ size at low concentration (Fig 5 and Table 3). When Isoflurane concentration was above 0.1% (v/v), however, EZ size diminished significantly and continued to diminish with further concentration increase. By 0.3% the EZ diminished to one third of its initial size.

**Table 3:** Effect of Isoflurane on EZ size.

Volume Isoflurane (μL)	Concentration (%)	Initial EZ (Normalized)	Mean	St. Dev.
0	0.0000	100.00	100.00	0.00
2	0.0999	100.00	130.11	5.71
4	0.1996	100.00	69.02	7.84
6	0.2991	100.00	41.24	1.56
8	0.3984	100.00	35.26	0.42
10	0.4975	100.00	31.39	2.29
12	0.5964	100.00	27.47	0.69

**Fig 6:**
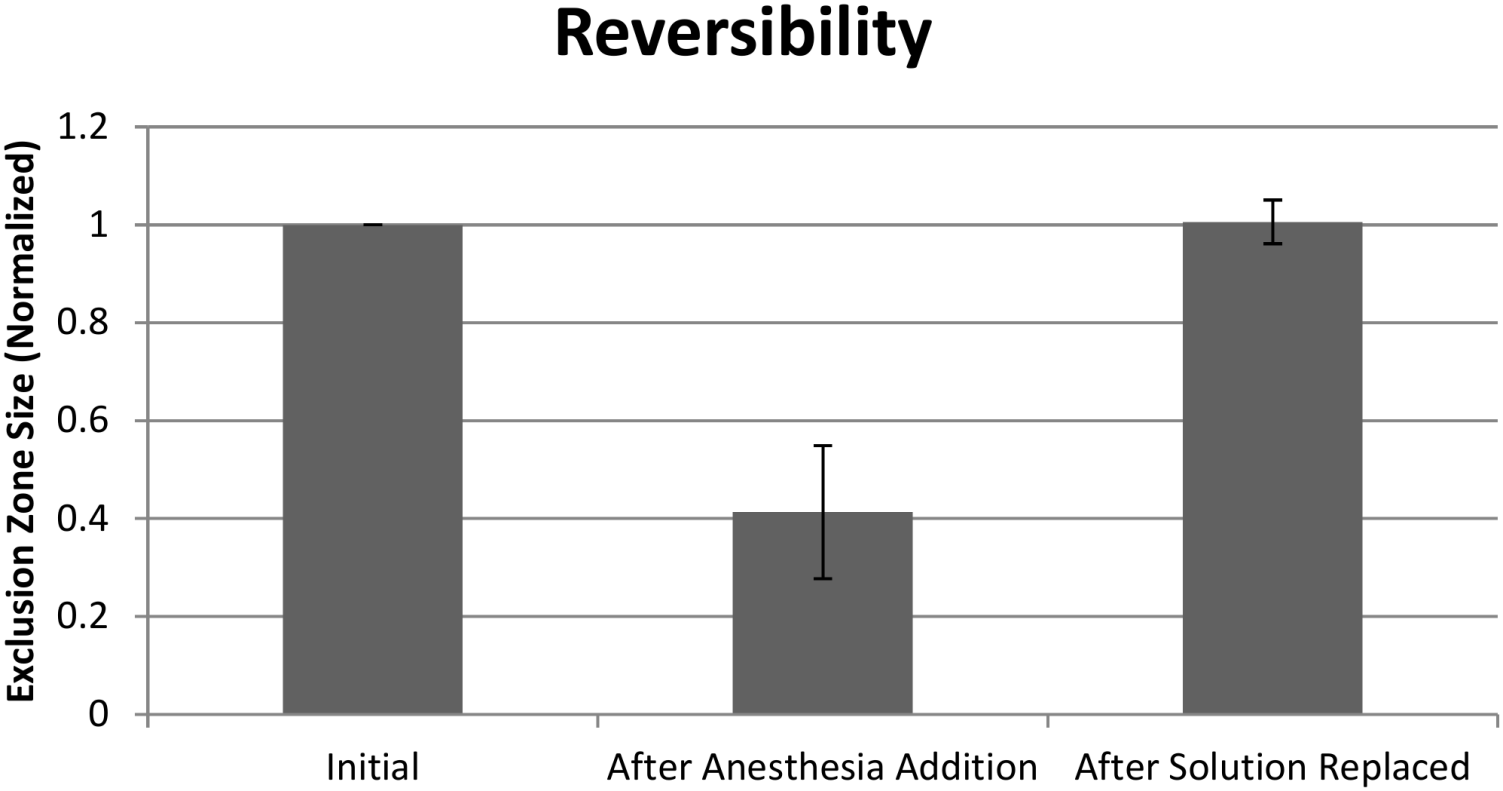
Reversibility results obtained with Bupivacaine. Full reversibility is demonstrated.

Fig 6 shows the reversibility of Bupivacaine’s effect. Once Bupivacaine was added, the exclusion zone diminished in size. After the microsphere solution was replaced with a control microsphere solution that contained no anesthetic, the exclusion zone returned to its original size, i.e., the size before the anesthesia was added. Similar results were obtained with Isoflurane.

## Discussion

The results show that anesthetics in clinically relevant concentrations have profound effects on interfacial water. We studied two local anesthetics, Lidocaine and Bupivacaine, as well as an inhalational anesthetic Isoflurane. All three anesthetics showed similar effects: at an extremely low concentration they expanded the zone of interfacial water (EZ). At higher concentrations they diminished EZ size in a concentration-dependent manner. The effects were fully reversible.

These effects follow common clinical experience. The extremely low anesthetic doses that patients experience during induction produce excitatory effects (known as Stage II or the “excitement” stage), while higher concentrations produce increasingly stronger levels of narcosis (known as Stages III and IV or the “surgical” and “overdose” stages). The question arises how these effects on interfacial water might help explain narcosis.

One possible explanation derives from the fact that cells are filled with interfacial water [13]. This (EZ) water bears negative charge [8]. Thus, at least some of the cell’s negative charge and negative electrical potential arise from EZ water. That electrical potential is evidently crucial for action potential generation, which bears responsibility for cellular communication and other cellular functions. If this intracellular potential is diminished through diminution of EZ water, then the cell becomes unable to properly function, and particularly unable to communicate. Proteins cannot fold, and currents cannot flow through membranes.

This result might help explain the observation that anesthetic-like inhibitory action occurs not only in animals with nerves but also in simple organisms and even single-celled creatures. In experiments conducted by Keil et al. [14], five different anesthetics were all found to inhibit respiration and meiosis in yeast cells, which lack nervous systems altogether. Similarly, when nematodes, which have very simple nervous systems, are exposed to anesthetics, the nematodes are initially “excited” and race around their environment until full narcosis sets in and they become unresponsive to external stimuli such as needle prods [15]. These experiments indicate that anesthetic-like effects extend to not just the advanced nervous systems of larger animals, but also to general biological functions that exist in more primitive species. The excitatory response at low dose is confirmed as well.

Anesthesia’s ability to affect the EZ’s size in cells can also explain how narcosis can be reversed by high pressure. Miller et al. describe how elevated atmospheric pressures antagonize the effects of both liquid and gaseous anesthetics [16]. It would seem that if diminished EZ caused narcosis, then increased pressure would need to increase the size of the EZ to weaken the anesthetic effect. Ypma and Pollack demonstrated that elevated pressure does in fact significantly increase the size of the EZ [17]. This result adds strength to the hypothesized relationship between narcosis and cellular water.

Thus, anesthetic action is a rather general effect that occurs widely across the phylogenetic spectrum. This generality squares with the results found here. Interfacial water is the most common substance in all cells. Water constitutes two thirds of cell volume; however, the water molecule is so small that numerous molecules are required to make up that two-thirds fraction. Thus, more than 99% of the cell’s molecules are water molecules. Hence, the most common molecule in the cell appears to be a common target of anesthetics. This finding does not preclude other sites of anesthetic action such as those generally accepted; however, it goes some way toward explaining the generality of the anesthetic effect.

## References

[1] Pollack GH. The Fourth Phase of Water: Beyond Solid, Liquid, and Vapor. Seattle, WA: Ebner and Sons; 2013.

[2] Meyer HH. Welche eigenschaft der anasthetica bedingt inre narkotische wirkung?. Arch Exp Pathol Pharmoakol. 1899; 42:111–113

[3] Overton CE. Studien über die narkose zugleich ein beitrag zur allgemeinen pharmakologie. Jena, Switzerland: Gustav Fischer; 1901

[4] Miller KW, Paton WD, Smith RA, Smith EB. The pressure reversal of general anesthesia and the critical volume hypothesis. Mol Parmacol. 1973; 9(2): 131–143. 4711696

[5] Koblin DD, Chortkoff BS, Laster MJ, Eger E, Halsey MJ, Ionescu P. Polyhalogenated and perfluorinated compounds that disobey the Meyer-Overton hypothesis. Anesth Analg. 79(6): 1043–1048. 7978424 10.1213/00000539-199412000-00004

[6] Franks NP, Lieb WR. Molecular mechanisms of general anaesthesia. Nature. 300(5892):487–493. 6755267 10.1038/300487a0

[7] Pauling L. A molecular theory of general anesthesia. Science. 7(134):15–21 10.1126/science.134.3471.15 13733483

[8] Zheng J, Chin W, Khijniak E, Khinjniak E Jr, Pollack GH. Surfaces and interfacial water: evidence that hydrophilic surfaces have long range impact. Advances in Colloid and Interface Science. 127: 19–27. 16952332 10.1016/j.cis.2006.07.002

[9] Wang HJ, Kleinhammes A, Tang P, Xu Y, Wu Y. Critical role of water in the binding of volatile anesthetics to proteins. 2013 Journal of Physical Chemistry. 117(40):12007–12012. doi: 10.1021/jp407115j 24050264 10.1021/jp407115j PMC3901425

[10] Gianelly R, von der Groeben JO, Spivack AP, Harrison DC. Effect of lidocaine on ventricular arrhythmias in patients with coronary heart disease. N Engl J Med 277:1215, 1967 4862377 10.1056/NEJM196712072772301

[11] Reynolds F. A comparison of the potential toxicity of bupivacaine, lignocaine and mepivacaine during epidural blockade for surgery. British J Anaesth 43:567, 1971. 10.1093/bja/43.6.567 5089935

[12] Katosj T, Suguro Y, Nakajima R, Kazama T, Ikeda K. Blood concentrations of sevoflurane and isoflurane on recovery from Anaesthesia. Br J of Anaesthesia, 69(3):259–62, 10/1992. 10.1093/bja/69.3.259 1389843

[13] Pollack GH. Cells, Gels and the Engines of Life: A New Unifying Approach to Cell Function. Seattle, WA: Ebner and Sons; 2001.

[14] Keil RL, Wolfe D, Reiner T, Peterson CJ, Riley JL. Molecular genetic analysis of volatile-anesthetic action. Mol Cell Biol. 1996 7; 16(7):3446–3453 8668160 10.1128/mcb.16.7.3446 PMC231339

[15] Morgan PG, Rajaram S, Spangler TL, Sedensky MM. (1999) “A stomatin and a degenerin interact to control anesthetic sensitivity in Caenorhabditis elegans”. Genetics. 1999 12; 153(4): 1673–1682. 10581275 10.1093/genetics/153.4.1673 PMC1460880

[16] Miller KW, Paton WDM, Smith RA, Smith EB. The pressure reversal of general anesthesia and the critical volume hypothesis. Molecular Pharmacology. 1973 3; 9(2) 131–143. 4711696

[17] Ypma RE, Pollack GH. Effect of hyperbaric oxygen conditions on the ordering of interfacial water. UHM 2015, 42(3) 257–264. 26152107

